# THE EFFECT OF GEROPSYCHOLOGY TRAINING ON JOB EFFECTIVENESS AMONG VA PSYCHOLOGISTS

**DOI:** 10.1093/geroni/igz038.2816

**Published:** 2019-11-08

**Authors:** Ashley Scales, Rachel Rodriguez, Christine Gould, Jeffrey Gregg, Joung Huh, Josea Kramer

**Affiliations:** 1 VA Palo Alto Health Care System, Palo Alto, California, United States; 2 Durham VA Health Care System, Durham, North Carolina, United States; 3 San Francisco VA Health Care System, San Francisco, California, United States; 4 VA Greater Los Angeles Health Care System, Sepulveda, California, United States

## Abstract

Providing training to assist employees with excelling in their job role may increase their job effectiveness, which then translates to improvements in an organizations’ performance measures. In 2018, the Geriatric Scholars Psychology Program measured whether a multi-day course influenced the Psychologists’ perceived job effectiveness using modified questions from Godat and Brigham’s Reaction Measure. Ninety-two percent agreed the training assisted them with identifying and overcoming obstacles; helped them set goals to increase problem solving at their designated facilities; boosted their confidence in fulfilling their job duties; and the training should be made available to other employees who provide care to older Veterans on a regular basis. Findings from the 3-month follow-up assessment demonstrated that the positive effect on job effectiveness was sustained. Discussion will explore aspects of the course that may be key in improving perceived effectiveness and consider novel approaches to enhance this outcome.

